# Mannose-binding lectin gene sequence data in Kelantan population

**DOI:** 10.1038/s41597-024-03274-4

**Published:** 2024-04-30

**Authors:** Muhamad Aidil Zahidin, Noor Haslina Mohd Noor, Muhammad Farid Johan, Abu Dzarr Abdullah, Zefarina Zulkafli, Hisham Atan Edinur

**Affiliations:** 1 Department of Haematology, School of Medical Sciences, Universiti Sains Malaysia (Health Campus), 16150 Kubang Kerian, Kelantan Malaysia; 2 Transfusion Medicine Unit, Hospital Universiti Sains Malaysia, 16150 Kubang Kerian, Kelantan Malaysia; 3 Department of Internal Medicine, School of Medical Sciences, Universiti Sains Malaysia (Health Campus), 16150 Kubang Kerian, Kelantan Malaysia; 4 Forensic Science Programme, School of Health Sciences, Universiti Sains Malaysia (Health Campus), 16150 Kubang Kerian, Kelantan Malaysia

**Keywords:** Genetics research, Diagnostic markers

## Abstract

The human mannose-binding lectin (*MBL*) gene encodes a polymorphic protein that plays a crucial role in the innate immune response. Human *MBL* deficiency is associated with immunodeficiencies, and its variants have been linked to autoimmune and infectious diseases. Despite this significance, gene studies concerning *MBL* sequencing are uncommon in Malaysia. Therefore, we aimed to preliminary described the human *MBL* sequencing dataset based on the Kelantan population. Blood samples were collected from 30 unrelated individuals and underwent DNA extraction, genotyping, and sequencing. The sequencing data generated 886 bp, which were deposited in GenBank (ON619541-ON619546). Allelic variants were identified and translated into six *MBL* haplotypes: HYPA, HYPB, LYPB, LXPB, HXPA, and LXPA. An evolutionary tree was constructed using the haplotype sequences. These findings contribute to the expansion of *MBL* information within the country, providing a valuable baseline for future research exploring the association between the gene and targeted diseases.

## Background & Summary

Human mannose-binding lectin (*MBL*) is a C-type serum lectin produced in the liver that plays a crucial role in innate immunity^1^. *MBL* may mediate phagocytosis by binding to specific carbohydrate moieties on various pathogens, thereby utilising identified phagocyte receptors^2^. Additionally, *MBL* employs *MBL*-associated serine proteases (MASP)-1 and −2 to activate the MBL pathway of complement^3^. The *MBL* protein is encoded by the polymorphic *MBL* gene, which consists of four exons interrupted by three introns and is located on chromosome 10 (10q11.2-q21)^4^.

Single nucleotide polymorphisms (SNPs) at specific nucleotide positions have been identified: −550 (G/C or *H*/*L*), −221 (C/G or *X*/*Y*), +4 (C/T or *P*/*Q*), +223 (C/T or *A*/*D*), +230 (G/A or *A*/*B*) and +239 (G/A or *A*/*C*) within the promoter/5′ untranslated and exon 1 regions^5^. Functional characterisation of SNPs in the promoter region, altering *MBL* transcription, underscores the significance of these genetic differences for *MBL* circulating levels and expression^6^. In the exon 1 region, the wild-type (normal) allele is denoted as *A*, while the *B* (codon 52), *C* (codon 54) and *D* (codon 57) allelic variants are collectively referred to as *O*. Changes in amino acid resulting from exon 1 variations are believed to influence the functional properties of the *MBL* protein collagenous region^7^.

Theoretically, 64 unique haplotypes could be generated by combining those six polymorphisms. However, only eight common haplotypes are typically observed in population studies due to significant linkage disequilibrium (LD) between the promoter and exon 1 SNPs^5,6^. Different haplotypes have been associated with high *MBL* levels, such as HYPA, LYQA, and LYPA; an intermediate *MBL* level represented by LXPA; and haplotypes linked to low *MBL* levels, including HYPD, LYPB, LYQC, and LYPD^8,9^.

Within the promoter and exon 1 regions, at least 30 SNPs and six deletion sites have been previously identified^10^. However, SNPs at positions −550, −221, +4, +223, +230, and +239 are commonly investigated and recognised as common point mutation secretors. These SNPs are frequently studied to characterise *MBL* variants and determine serum *MBL* protein levels. This strategy can be implemented using either serum level measurements or genotyping method such as polymerase chain reaction-sequence-specific amplification (PCR-SSP), -sequence-based typing (PCR-SBT), -restriction fragment length polymorphism (PCR-RFLP) or direct sequencing. Previous studies have shown that these *MBL* SNPs are associated with autoimmune diseases such as rheumatoid arthritis (RA)^11,12^, Sjögren syndrome (SS)^13,14^ and systemic lupus erythematosus (SLE)^15,16^; as well as infectious diseases including pulmonary tuberculosis (TB)^17,18^, acute respiratory infection (ARI)^19,20^ and vulvovaginal candidiasis (VVC)^21,22^.

**Fig. 1:**
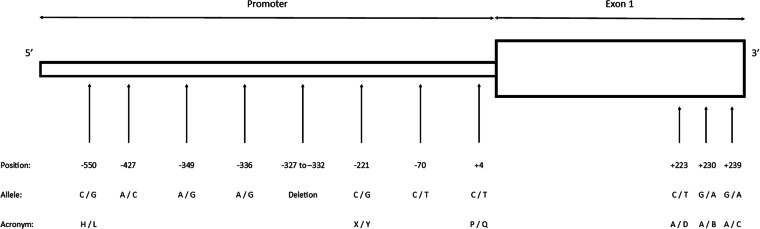
Location of SNPs’ point mutation on the promoter and exon 1 of *MBL* gene. Noted that the figure is not drawn to scale.

**Fig. 2:**
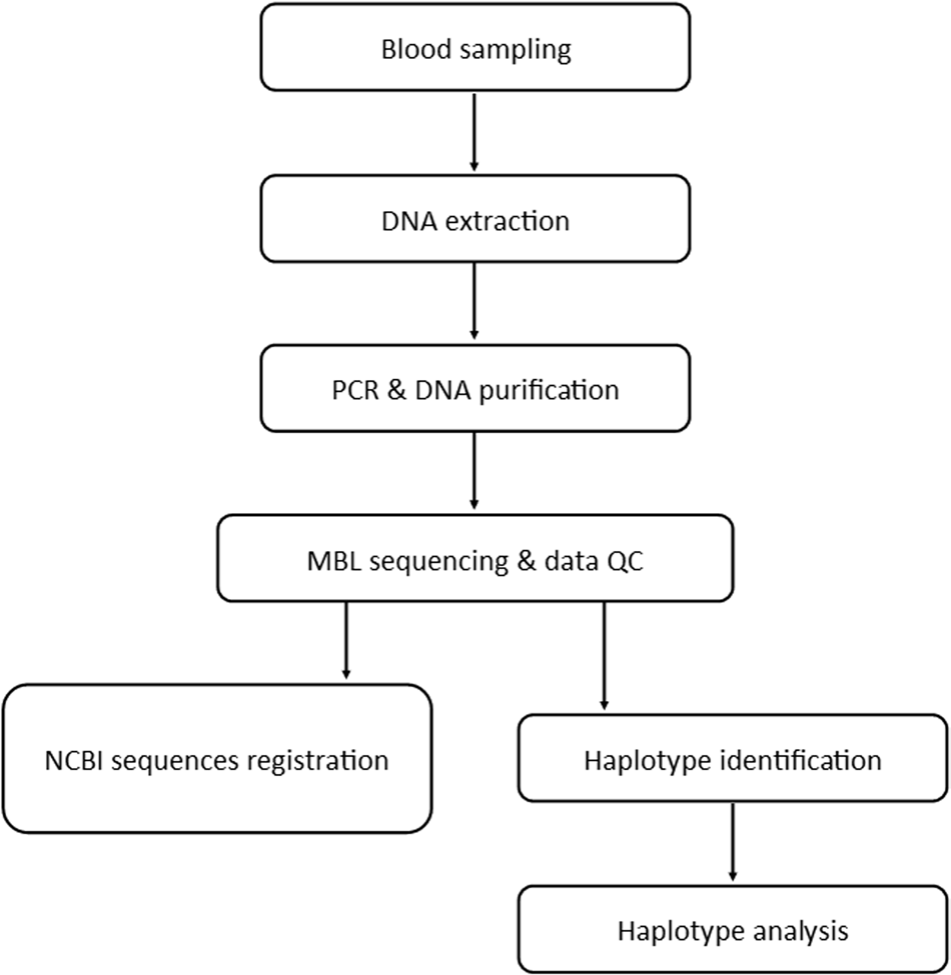
Workflow of research study. QC- Quality control, NCBI- National Centre for Biotechnology Information.

Despite a wide coverage of studies and a flux of research findings, studies on the *MBL* gene in Malaysia are uncommon. Here we preliminary described the human *MBL* sequencing datasets, which include six common point mutation secretors and five additional polymorphic sites in the promoter region among Kelantan individuals (Table 1, Fig. 1). A diagram illustrating the workflow in this study is presented in Fig. 2. Genomic DNA was extracted from blood samples and subjected to direct PCR and Sanger sequencing. The resulting 886 bp sequence products were assembled using molecular genetic software to identify *MBL* haplotypes, which were later used to construct an evolutionary tree. This preliminary study provides baseline information for future research on the association between *MBL* gene polymorphisms and targeted diseases. Additionally, the generated data will be representative of the Malaysian population and may encourage broader population studies in the future.

**Table 1:** The profile of *MBL* polymorphic sites at chromosome 10q11.2.

	Location	SNP	rs	Allele	Acronym
1	Promoter	−550	11,003,125	G → C	H → L
2	Promoter	−427	11,003,124	A → C	
3	Promoter	−349	7,084,554	A → G	
4	Promoter	−336	36,014,597	A → G	
5	Promoter	−327 to −332	10,556,764	Del	
6	Promoter	−221	7,096,206	C → G	X → Y
7	Promoter	−70	11,003,123	C → T	
8	Promoter	+4	7,095,891	C → T	P → Q
9	Exon 1, Codon 52	+223	5,030,737	C → T	A → D
10	Exon 1, Codon 54	+230	1,800,450	G → A	A → B
11	Exon 1, Codon 57	+239	1,800,451	G → A	A → C

SNP- single nucleotide polymorphisms, rs- Reference SNP, Del- deletion.

## Methods

### Ethical statement

This study was approved by the Human Research Ethics Committee of Universiti Sains Malaysia (USM/JEPeM/19090533) and was performed in accordance with the guidelines set forth by the National Blood Centre, Ministry of Health Malaysia. Signed informed consent and demographic background information were obtained from each participant who agreed to take part in the study, and the data will be published without revealing their identities.

### Sample collection and DNA extraction

Genomic samples were collected from 30 unrelated individuals from Kelantan. Approximately, 10 cc of blood was drawn from the peripheral vein and stored in the ethylenediaminetetraacetic acid (EDTA) tube. The total DNA was extracted using the gSYNC^TM^ DNA Extraction Kit (Geneaid, Taiwan), following protocol provided by the manufacturer.

### *MBL* genotyping

The isolated genomic DNA was amplified using a set of forward reverse primers specific for *MBL* (Table 2)^19^. The PCR amplification was carried out using Veriti TM 96-well fast thermal cycler. Subsequently, the amplified PCR products were purified using the GeneJET PCR Purification Kit (Thermo Scientific, United States). DNA sequencing was performed using ABI 3100 DNA Sequencer at First Base Laboratories Sdn Bhd (Malaysia).

**Table 2:** Properties of *MBL* primers used.

Primer	Sequence (5′ - 3′)	Length	Tm	GC%	Product length
Forward	CCT GCC AGA AAG TAG AGA GG	20	57.02	55.00	954
Reverse	CCA GGC AGT TTC CTC TGG AAG G	22	62.85	59.09	

### Sequencing analysis

**Fig. 3:**
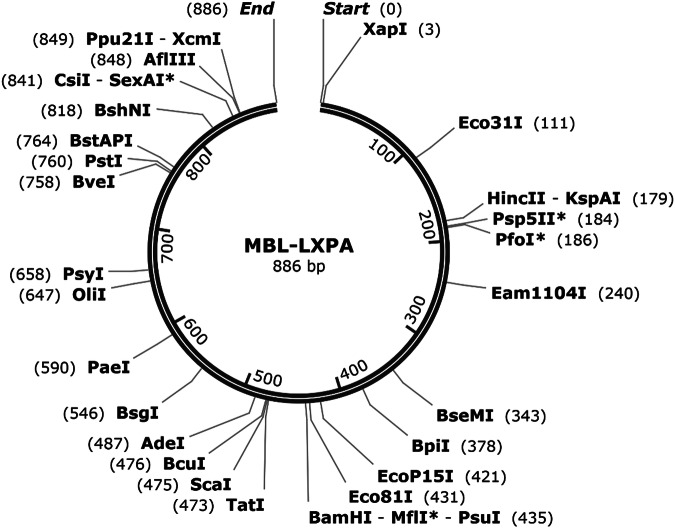
Map of 884 bp *MBL* sequence.

**Fig. 4:**
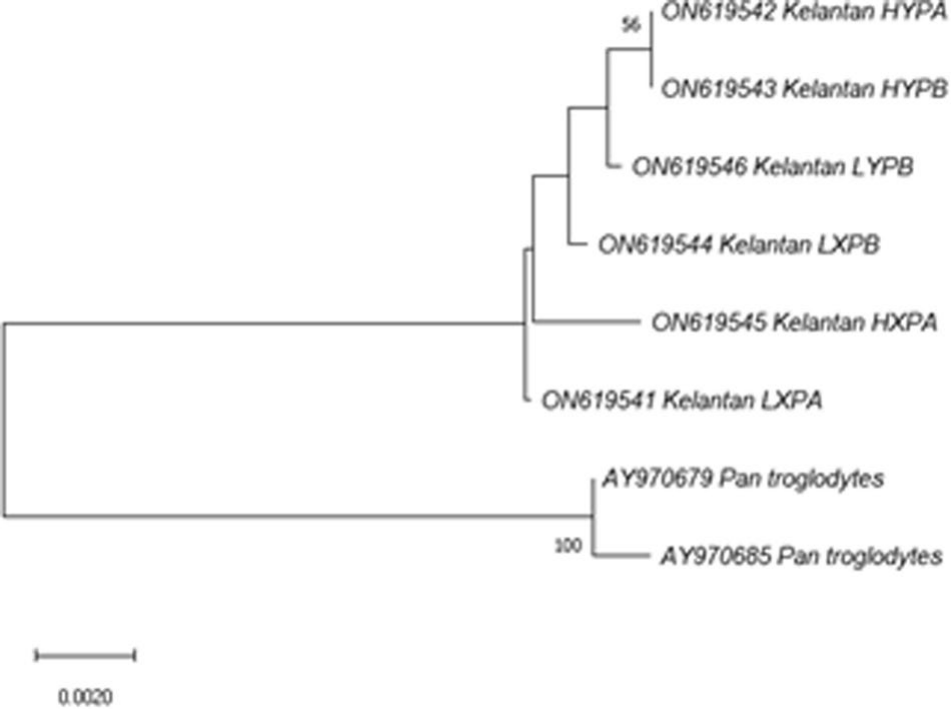
Neighbor-joining (NJ) tree of *MBL* in this study. The evolutionary distances were computed using Kimura 2-parameter method.

The raw sequences were visualised and analysed using SnapGene version 6.1 (Fig. 3). Subsequently, all sequences were aligned with the *Homo sapiens MBL2* RefSeqGene (LRG_154) of chromosome 10, utilising Sequencher version 5.4.6. The identification of *MBL* haplotypes was based on the allelic mutations (Table 1)^5^. Additionally, a Neighbor-joining tree^23^ was constructed using the Molecular Evolutionary Genetics Analysis (MEGA) version 11^24^, with *Pan troglodytes* (chimpanzee) sequences (AY970679 and AY970685)^25^ as an outgroup (Fig. 4).

## Data Records

The sequencing data are accessible in the National Centre for Biotechnology Information (NCBI), assigned with the reference numbers ON619541-ON619546 (Table 3)^26–31^. These sequencing information details pertain to a subset of individuals.

**Table 3:** List of registered *MBL* haplotypes in the NCBI website.

	Haplotype	GenBank accession number
1	LXPA	ON619541
2	HYPA	ON619542
3	HYPB	ON619543
4	LXPB	ON619544
5	HXPA	ON619545
6	LYPB	ON619546

## Technical Validation

The *MBL*-PCR products underwent assessment to confirm the absence of contamination through 2% gel electrophoresis. Subsequently, the raw sequences were aligned with RefSeqGene (NG_008196), and each sequence was manually inspected to ensure the absence of three stop codons: UAA, UAG and UGA. The evolutionary history was inferred using the NJ method^23^. The percentage of replicate trees (>50%) in which the associated taxa clustered together in the bootstrap test (1,000 replicates) I indicated next to the branches^32^.

## Data Availability

The analyses were conducted using the version and parameters as described below: 1) SnapGene, version 6.1, parameters used: interrupted circle, show polymorphism cite locations, unique 6+ cutters 2) Sequencher, version 5.4.6, parameters used: minimum match percentage 85, minimum overlap 20. 3) MEGA, version 11^24^, parameters used: NJ tree with Kimura2 parameter model^33^.
