# Author Correction: Mannose-binding lectin gene sequence data in Kelantan population

**DOI:** 10.1038/s41597-024-04334-5

**Published:** 2025-01-07

**Authors:** Muhamad Aidil Zahidin, Noor Haslina Mohd Noor, Muhammad Farid Johan, Abu Dzarr Abdullah, Zefarina Zulkafli, Hisham Atan Edinur

**Affiliations:** 1 Department of Haematology, School of Medical Sciences, Universiti Sains Malaysia (Health Campus), 16150 Kubang Kerian, Kelantan Malaysia; 2 Transfusion Medicine Unit, Hospital Universiti Sains Malaysia, 16150 Kubang Kerian, Kelantan Malaysia; 3 Department of Internal Medicine, School of Medical Sciences, Universiti Sains Malaysia (Health Campus), 16150 Kubang Kerian, Kelantan Malaysia; 4 Forensic Science Programme, School of Health Sciences, Universiti Sains Malaysia (Health Campus), 16150 Kubang Kerian, Kelantan Malaysia

**Keywords:** Genetics research, Diagnostic markers, Genetics research, Diagnostic markers

Correction to: *Scientific Data* 10.1038/s41597-024-03274-4, published online 30 April 2024

In the acknowledgements section the grant number ‘FRGS/203/PPSP/6171241’ was incorrect and should have read ‘FRGS/1/2019/SKK06/USM/02/6’. The original article has been corrected.

